# Braille recognition by E-skin system based on binary memristive neural network

**DOI:** 10.1038/s41598-023-31934-9

**Published:** 2023-04-03

**Authors:** Y. H. Liu, J. J. Wang, H. Z. Wang, S. Liu, Y. C. Wu, S. G. Hu, Q. Yu, Z. Liu, T. P. Chen, Y. Yin, Y. Liu

**Affiliations:** 1 State Key Laboratory of Electronic Thin Films and Integrated Devices, University of Electronic Science and Technology of China, Chengdu, 610054 People’s Republic of China; 2 School of Integrated Circuits, Guangdong University of Technology, Guangzhou, 510006 China; 3 School of Electrical and Electronic Engineering, Nanyang Technological University, 50 Nanyang Avenue, Singapore, 639798 Singapore; 4 Graduate School of Engineering, Gunma University, 1-5-1Tenjin, Kiryu, Gunma 376-8515 Japan; 5 Deepcreatic Technologies Ltd, Chengdu, 610000 Sichuan People’s Republic of China

**Keywords:** Electrical and electronic engineering, Electronic devices

## Abstract

Braille system is widely used worldwide for communication by visually impaired people. However, there are still some visually impaired people who are unable to learn Braille system due to various factors, such as the age (too young or too old), brain damage, etc. A wearable and low-cost Braille recognition system may substantially help these people recognize Braille or assist them in Braille learning. In this work, we fabricated polydimethylsiloxane (PDMS)-based flexible pressure sensors to construct an electronic skin (E-skin) for the application of Braille recognition. The E-skin mimics human touch sensing function for collecting Braille information. Braille recognition is realized with a neural network based on memristors. We utilize a binary neural network algorithm with only two bias layers and three fully connected layers. Such neural network design remarkably reduces the calculation burden and, thus, the system cost. Experiments show that the system can achieve a recognition accuracy of up to 91.25%. This work demonstrates the possibility of realizing a wearable and low-cost Braille recognition system and a Braille learning-assistance system.

## Introduction

Individuals who are blind or visually impaired (BVI) encounter much inconvenience in life, especially information exchange like reading and writing. The Braille system, invented in 1824, is widely used as an important and effective means of communication for BVI. The six-dot Braille system consists of three rows and two columns of raised dots, with different combinations of raised dots used to represent different characters. However, learning Braille is a challenging process for BVI. In addition, the current Braille reading system has some disadvantages, such as high cost, large size and lack of portability. Therefore, it would be meaningful to realize a portable and low-cost intelligent Braille recognition system as an auxiliary tool to help BVI recognize Braille or learn Braille efficiently.

With the availability of flexible materials and sensors, flexible sensing under various stimulations (e.g., pressure) and environmental conditions (e.g., temperature, humidity, etc.) has been demonstrated^1,2^. Some sensors were used for the detection of human joint activity, muscle movement and Breathing condition^3–7^. Meanwhile, the combination of flexible sensing and artificial intelligence (AI) technology could be used to mimic the human touch sensing function^8–11^. A few of works have reported the AI-based Baillie recognition with Graphical Processing Unit (GPU)^10,11^. There are also some works related to Braille identification^12,13^. However, such designs were typically realized with GPUs, which were costly and energy-consuming. Memristor has been used in artificial neural networks because of its similarities to synapses in the human brain^14–17^. The complexity and power consumption of artificial neural networks with memristors can be significantly reduced by mapping the weights of a network to the memristors^18–20^. Although Static Random-Access Memory (SRAM) is also often used to map the weights of neural network algorithms, for board-level circuits, common SRAM chips usually only have digital interfaces and can only process digital signals. The SRAM-based board-level system requires peripheral circuits like comparators and addition trees. Completing a multiply accumulate (MAC) operation on SRAM-based system requires multiple cycles, increasing the control program's complexity. The memristor-based system in this work supports analog-domain computing, and the MAC operation only needs to add an operational amplifier at the bottom of the array, and the calculation can be completed in one computing cycle. At present, there are also some researches on SRAM that can support multi-row parallel computing, but most of them are calculated in the analog domain, and special peripheral circuits are required, which is not easy to implement in board-level circuits. The combination of flexible sensing and memristor-based artificial neural networks provides the possibility of realizing a portable and low-cost Braille recognition system.

In this work, the strategy of using the combination of flexible sensing and a memristor-based artificial neural network was implemented to realize Braille recognition. Polydimethylsiloxane (PDMS)-based flexible pressure sensors were fabricated to construct an electronic skin (E-skin) for flexible sensing. Although some pressure sensors with higher sensitivity and better robustness have emerged, they require more materials or more complex fabrication processes, such as the preparation of thin films with microstructures^6,7^. This work only requires the pressure sensor to detect whether the raised point of the Braille is pressed. The PDMS-based pressure sensor is sufficient to meet the system requirements, so the low-cost and easy-to-fabricate PDMS-based pressure sensor was chosen. On the other hand, HfO_2_ memristor devices, which have the capability of being used as synapses to store weights in artificial neural networks and the advantages of being non-volatile and can reduce system complexity, were used to implement a Binary Artificial Neural Network (BANN) for intelligent Braille recognition. The relevant information about the HfO_2_ memristor devices used in this work was reported in our previous works^18,21,22^. The artificial neural network has two bias layers and three fully connected layers. Such a design remarkably reduces the usage of computing resource and, thus, the system cost. The experimental result shows that the system can achieve a recognition accuracy of up to 91.25%.

## Method and experiment

**Figure 1:**
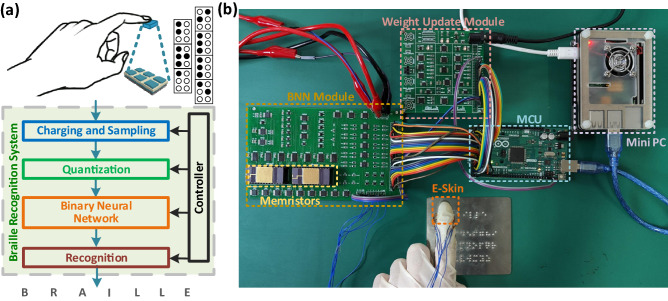
(**a**) Illustration of operation of the intelligent Braille recognition system consisting of the E-skin and memristor-based BANN; and (**b**) photograph of the hardware of the system.

Figure 1a illustrates the operation of the intelligent Braille recognition system consisting of the E-skin and memristor-based BANN, and Fig. 1b shows a photograph of the hardware of the system. The BANN module and weight update module in the system were mainly used for the weight mapping during the inference stage of the Braille recognition as well as charging and sampling of the capacitive electronic skin. The Micro Controller Unit (MCU) was responsible for controlling the system, while the Raspberry Pi Mini-PC was used for data visualization and control of the weight update module. As shown in Fig. 1b, the E-skin was attached to a human finger for tactile sensing which was realized based on the detection of change in the capacitance of the touch sensors in the E-skin. The electrical signal (i.e., the change in the capacitance) of tactile sensing was served as the stimulus to the neurons of the BANN. The memristors acted as synapses between neurons to map the weights of the BANN. The Binary Fully Connected (B-FC) neural network model was employed for Braille recognition.

1. Fabrication of PDMS-based E-Skin

**Figure 2:**
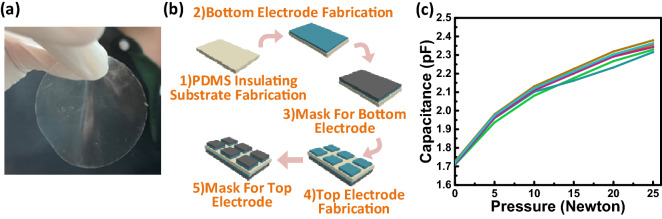
(**a**) Photograph of the standalone PDMS layer; (**b**) process flow of fabrication of the flexible E-skin; and (**c**) a typical relationship between the applied pressure and capacitance for a capacitive sensor.

The E-skin was fabricated with the formation of capacitive touch sensors on a thin PDMS membrane dielectric layer. The PDMS layer was first formed on a silicon wafer. To enable easy removal of the PDMS layer from the wafer, a thin layer of photoresist was deposited on a cleaned wafer as the sacrificial layer and the wafer was baked at 100 °C for 2 min. PDMS monomer and curing agent were mixed at a ratio of 10:1. The mixed solution was spin-coated on the as-prepared wafer using a KW-4A spin coater (Chemat Technology) with a rotational speed of 500 rpm for 2 min. Then the wafer was immersed in acetone solution for 2–3 min to dissolve the photoresist, and finally a standalone PDMS layer with a thickness of about 100 µm was fabricated. Figure 2a shows a photograph of the PDMS layer. Conductive silver paste was printed on both sides of the PDMS layer to form the six top electrodes with the dimensions 1.7 mm × 1.7 mm and the bottom electrode with the dimensions 6.5 mm × 3.5 mm of the capacitive touch sensors. In order to make the capacitance change more obvious, a non-conductive hard mask is added to the electrodes. An E-skin with a 3 × 2 capacitive sensor array was fabricated for sensing the six points of Braille. The process flow of the E-skin fabrication is shown in Fig. 2b.

In the touch sensing experiment, an Arduino UNO Micro Controller Unit (MCU) and a MPR121 proximity capacitive touch sensor controller (Freescale Semiconductor) were used to detect the change in capacitance when the sensors were pressed. A digital force gauge (model SF-30N) was used to measure the applied pressure. A typical relationship between the applied pressure and capacitance for a capacitive sensor is shown in Fig. 2c.

**Figure 3:**
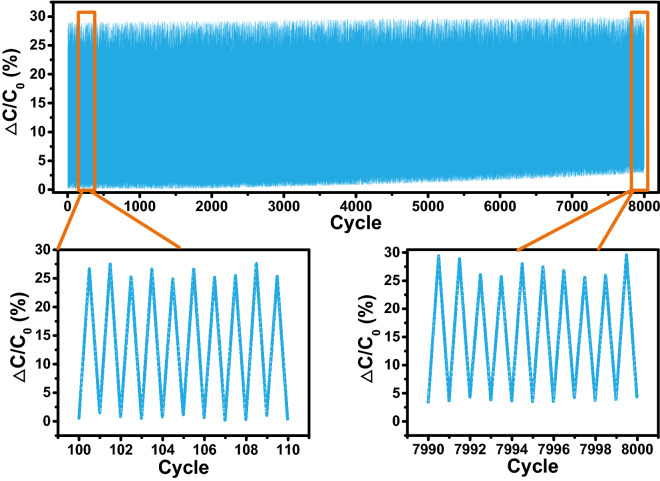
Reliability test of the fabricated sensor under the repeated 8000 compression-release cycles.

As shown in Fig. 3, we conducted a repeated compression-release test on the sensor for 8000 cycles under a pressure of about 10 Newton. For the Braille recognition system in this work, the capacitance change produced by the 10 Newton pressure on the sensor is enough to determine whether the sensor is pressed to the Braille. C0 represents the initial capacitance value of the sensor, and ΔC represents the change in the sensor capacitance value. As the number of compression-release cycles increases, the capacitance value of the sensor under the condition of no compression has a certain deviation from C0, and the deviation reaches 4% at 8000 cycles. We treat this variation as noise in the input to the system and address it by adding random noise to the input when training the braille recognition algorithm.

(2) Characterization of Memristors

**Figure 4:**
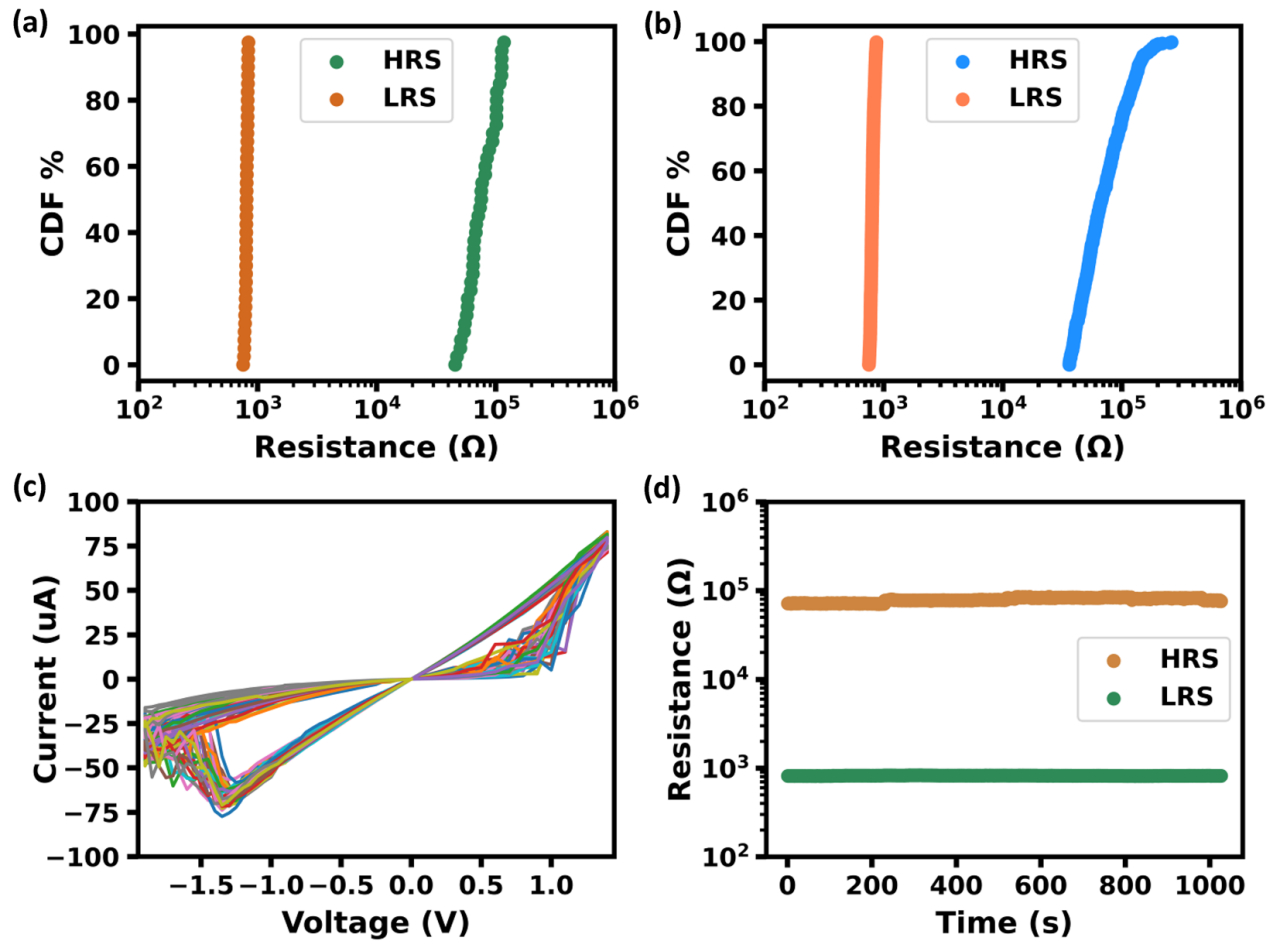
(**a**) Cumulative distribution function (CDF) of resistance of the high resistance state (HRS) and low resistance state (LRS) of 40 memristors; (**b**) typical CDF of cycle-to-cycle variation of both HRS and LRS of a memristor (500 cycles); (**c**) typical hysteresis characteristics of a memristor for 50 repeated measurements; and (**d**) typical retention characteristic of a memristor.

The relevant information about the HfO_2_ memristor devices used in this work was reported in our previous works^21,22^. The memristor used in this work has good endurance and can be programmed more than 10,000 times^22^. We selected 40 memristors to build the hardware platform. In order to map weight of the algorithm, a tightening of the low-resistance state (LRS) distribution (0.75–0.87 KΩ in this work) is required. To meet the requirements, an iterative write-verify scheme is used to set memristors. First, for an HRS memristor, we apply a positive pulse with an initial amplitude of 1 V for ~ 100 μs to set it to the LRS. If the resistance of the memristor is within the target resistance range of the LRS, the process is complete. If the resistance value is higher than the target range of LRS, we gradually increase the amplitude of the positive pulse applied to the memristor in steps of 0.05 V, until the resistance value reaches the required value. If the resistance value after applying the positive pulse is lower than the minimum value of the target range, a ~ 100 μs negative pulse with an amplitude of 0.5 V is applied to the memristor. If the resistance is still lower than the minimum value of the target range after applying the negative pulse, we gradually increase the amplitude of the negative pulse applied to the memristor in steps of 0.05 V, until the resistance value reaches the requirement. If the resistance is higher than the maximum value of the target range after applying the negative pulse, the memristor is reset to HRS and the process is repeated from the first step. By repeating the previous method within 10 times, the LRS resistance of the selected 40 memristors can all be within the target LSR range. Figure 4a shows the cumulative distribution function (CDF) of resistance of the high resistance state (HRS) and low resistance state (LRS) of the 40 memristors, and Fig. 4b shows the typical cycle to cycle characteristics of a memristor for 500 cycles. As shown in the figures, the resistance ratio between the HRS and LRS was larger than 40. The selected memristors had LRS resistance within the range of 0.75–0.87 KΩ, while their HRS resistance was larger than ~ 36 KΩ. Figure 4c shows typical hysteresis characteristics of a memristor for 50 repeated voltage sweeping in the voltage range of − 1.85 to + 1.4 V. As demonstrated in Fig. 4d, both the HRS and LRS exhibited a good retention capability, which meeting the requirements of the BANN.

(3) Binary Braille Recognition Algorithm

**Figure 5:**
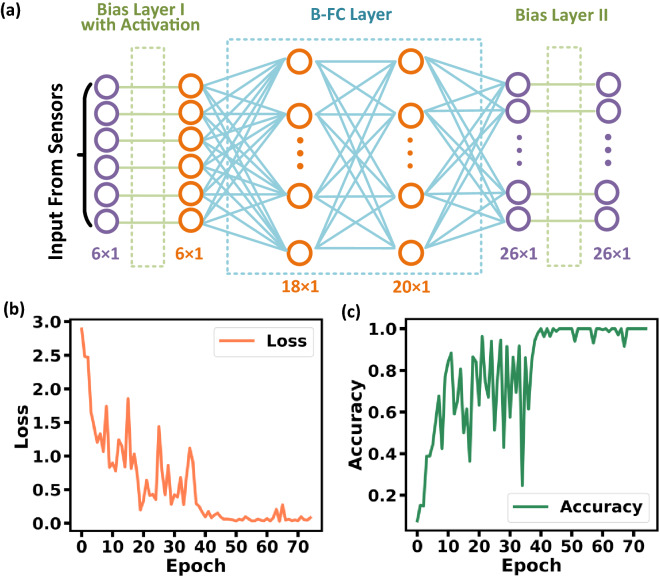
(**a**) Schematic illustration of the BANN structure; (**b**) the loss and (**c**) accuracy of the BANN as a function of number of epochs during the training process.

The Braille recognition was realized using a BANN algorithm. The neural network consisted of two bias layers and three fully connected (FC) layers as shown in Fig. 5a. The weight matrix of the BANN was trained with a GPU (Nvidia RTX 3080Ti). The training process was carried out in the python-based PyTorch™ framework, and back-propagation (BP) algorithm and Adaptive Moment Estimation (ADAM) optimizer were used to train the BANN. To achieve a better accuracy in the training with the test set, we adopted the methods of dynamic learning rate and early stop in training.

In order to train the BANN using the back-propagation algorithm, a continuously differentiable activation function (Eq. (1)) was used, and a 32bit Floating Point (FP32) version of weights with a range from − 1 to 1 were stored and updated in the training process.

1 $$H\left( x \right) = \left\{ \begin{gathered} - 1\quad \quad \quad \;\;\;x < - 1 \hfill \\ x^{2} + 2x\quad \;\; - 1 \le x < 0 \hfill \\ - x^{2} + 2x\quad 0 \le x < 1 \hfill \\ 1\quad \quad \quad \quad \;\;1 \le x \hfill \\ \end{gathered} \right.$$

Figure 5b and c show the training process. As can be observed, after around 50 epochs, the BANN achieved more than 90% recognition accuracy.

After the training, the previously used activation function was replaced with the following sign function.

2 $$\begin{array}{*{20}c} {x^{b} = Sign\left( x \right) = \left\{ {\begin{array}{*{20}c} { + 1 \;if\,\, x \ge 0 } \\ { - \,1 \,\,otherwise} \\ \end{array} } \right.} \\ \end{array}$$

During the inference process of Braille recognition, the weights were quantized with Eq. (2). The lightweight binary algorithm had a minimal number of network parameters, which saved significant energy and hardware cost. During training, we added 10% random noise to the input to make the Braille recognition algorithm model more robust.

(4) Mapping of Braille Recognition Algorithm

**Figure 6:**
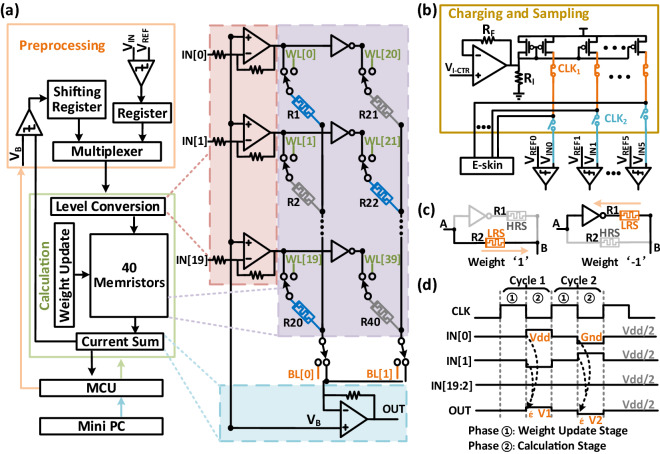
(**a**) Block diagram of the Braille recognition system based on the memristive neural network; (**b**) the circuit schematic of the charging and sampling module; (**c**) schematic of the circuit used to realize positive and negative weights; and (**d**) waveform diagram of the operation of the calculation module.

Figure 6a shows the block diagram of the Braille recognition system, which consists of the preprocessing module, calculation module, MCU and Mini PC. The preprocessing module was used for processing the Braille signals (V_IN_), selecting the data to be sent to the calculation module and registration of intermediate results from the calculation module. The memristor-based calculation module was responsible for the weight update and MAC operation. The MCU was used to provide the clock, control signals and reference signals. The acquisition of Braille signals was performed by the charging and sampling module. The circuit schematic of the charging and sampling module is shown in Fig. 6b. The charging and sampling module had a current source controlled by V_I-CTR_ and R_I_, used to charge the touch sensor array. The voltage of the sensor array is the system input. The switching between the charging and sampling paths was controlled by two complementary clocks of CLK_1_ and CLK_2_.

The preprocessing module had six comparators. The reference voltages (V_REF0_–V_REF5_) for the six comparators were provided by the digital to analog converter (DAC) controlled by the MCU. The signals, V_IN0_–V_IN5_ from the sensor array were compared with the reference voltages to map the first bias layer with activation, and the result was stored in the register. As shown in Fig. 6c, the memristor served as a synapse for storing weights. Each weight was represented using two memristors in complementary states to map the weights of + 1/− 1. As shown in left part of Fig. 6c, when the input terminal “A” was “+1”, memristor R1 was in the HRS and memristor R2 was in the LRS, indicating a weight of “ + 1”. The current through HRS was much lower than that through LRS, resulting in the output terminal “B” being “1”. The current through R2 was dominant and charges terminal "B". When the input terminal “A” was “+1” and the weight was “− 1” as shown in the right part in Fig. 6c, the current through R1 is dominant and discharges terminal "B". It can be observed that the output corresponds to the “XNOR” of the input and the weight.

The waveform diagram of the MAC operation performed by the calculation module is shown in Fig. 6d. The calculation module completed a MAC operation of input and weight in each calculation cycle, which included the weight update stage and the calculation stage. During the weight update stage, the memristor was connected to the weight update module through WL[39:0] and BL[1:0], and the Mini-PC and MCU controlled the weight update module to write weights. The weight update module could sequentially update the weight of each memristor by using the multiplexer (MUX) to select the required WL and BL. During the calculation stage, the memristors were connected as shown in Fig. 6a to perform the MAC operation. The calculation module enabled parallel processing of up to 20 inputs (IN[19:0]). Figure 6d shows the change of output points (OUT) when IN[1:0] were valid and IN[19:2] were invalid. As IN[19:2] were set to Vdd/2, the same value as V_B_, they had no effect on the output. In cycle 1, IN[0] was set to Vdd to represent input “+ 1”, and IN[1] was set to Gnd to represent input “− 1”. Memristor R1 was in the LRS and memristor R21 was in the HRS, indicating a weight of “+ 1”. Memristor R22 was in the LRS and memristor R2 was in the HRS, indicating a weight of “− 1”. The voltage change in OUT, denoted by ΔV1, was equal to twice the smallest incremental voltage, was the voltage change in OUT resulting from a single weight of "1" multiplied by the input "1" (as shown in Fig. 6a). In cycle 2, the weight remained unchanged, while IN[0] and IN[1] were set to Gnd and Vdd respectively. The difference (ΔV2) between the voltage of OUT and Vdd/2 satisfies Eq. (3),

3 $$\begin{array}{*{20}c} {\Delta V2 = - \Delta V1} \\ \end{array}$$

**Figure 7:**
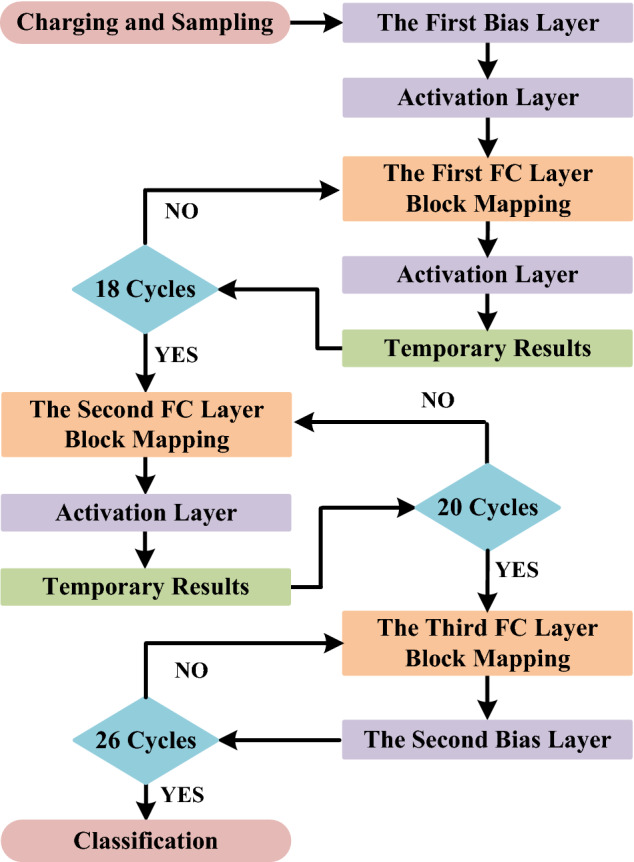
Flowchart of the binary memristor-based Braille recognition operation.

The mapping flow chart of the memristor-based Braille recognition algorithm is shown in Fig. 7. After the preprocessing module had processed the Braille signals (refer to Fig. 6a), data in the register were sent to the calculation module by the MUX as the input data of the first FC layer. In this stage, 6 × 2 memristors were involved in the calculation. The mapping of the first FC layer required 18 computation cycles to calculate the MAC of 18 neurons, and the MUX selected the data in the register as the input of the calculation module. Each computation cycle included a weight update stage and a computation stage. Since the limit of the read voltage of a memristor used in this work was about ± 0.5 V, a Level Conversion module was introduced to limit the voltage applied to the memristor. The power supply voltage of the inverters in the memristor array was Vdd/2 ± 0.5 V. In the computation stage, the Current Sum module output the MAC result, which was then compared with V_B_ (= Vdd/2) to complete the mapping of the activation layer. The result of each computation cycle was serially input into the shift registers. After the mapping of the first FC layer with activation was completed, the calculation results were sequentially stored in the shift register module.

When mapping the second FC layer, the MUX took the 18-bit intermediate data stored in the shift register as input and sent it into the calculation module in parallel. In this stage, 18 × 2 memristors participated in the calculation. It took 20 cycles to map the second FC layer. The intermediate results of the calculation were also stored in the shift register. The mapping of the third fully connected layer required 26 computation cycles. The result of each cycle was sent to the Analog to Digital Converter (ADC) module of the MCU for post-processing to complete the mapping of the second bias layer. Finally, the MCU selected the maximum value from the 26 results to determine the recognition result.

### Participants

This work proposed a portable low-cost braille recognition system based on memristors, focusing on the task of implementing braille recognition on a lightweight hardware platform. There were no people who were blind or visually impaired among the participants. Twenty males (mean age = 24, range 20–28) participated in the experiment. All participants were right-handed and had normal or corrected to normal vision. The participants were students or college graduates with a minimum of 4 years of higher education (mean = 6 range 4–9 years of education). All participants, unfamiliar with Braille, used the E-skin Braille recognition system proposed in this work to press the Braille printing plate to collect the Braille signals for neural network model training and complete the Braille recognition test. The Ethics Committee of University of Electronic Science and Technology of China approved the current study. An informed consent and a consent to publish were obtained from each of the participants. Confirming that all experiments were performed in accordance with relevant guidelines and regulations.

## Results and discussion

The Braille data set used for training and validating the algorithm was collected by 20 persons using the E-Skin in this work. The total data set contained 10,000 examples, including a random amount of 26 letters. 7000 samples randomly selected from the dataset were used for training, 2000 samples were used for verification, and 1000 sets of data were used for examination.

The mapping of the weights of the algorithm to the memristors and the characteristics of the memristors are shown in Figs. 4 and 6c, respectively. In Fig. 6c, when point A was set to V_READ_, the ideal charging/discharging current (I_IDEAL_) of point B should be V_READ_/R_LRS_, where V_READ_ was ± 0.5v in this work, and R_LRS_ represented the low resistance value of the memristor. However, due to the resistance variation of the memristors, there would be errors in the charging/discharging currents. The resistance of LRS of the selected memristors in this work had an average value of 0.81KΩ and variation of ± 7.4%, while the resistance of HRS was more than 40 times that of LRS. Therefore, the range of the charging/discharging current (I) can be expressed as follows,

4 $$\begin{array}{*{20}c} {\frac{{V_{READ} }}{{R_{LRS\_Max} }} - \frac{{V_{READ} }}{{R_{HRS} }} < I < \frac{{V_{READ} }}{{R_{LRS\_Min} }}} \\ \end{array}$$

where R_LRS_Max_ and R_LRS_Min_ represented the maximum and minimum resistance values of LRS, respectively, and R_HRS_ represented the resistance value of HRS. According to the range of resistance values for LRS and HRS of the memristors used in this work, R_LRS_Max_, R_LRS_Min_, and R_LRS_ were 0.87, 0.85, and 0.81 k respectively, and the value of R_HRS_ was 40 times greater than R_LRS_. Therefore, Eq. (4) can be rewritten as Eq. (5)

5 $$\begin{array}{*{20}c} {\frac{{V_{READ} }}{{1.074 \times R_{LRS} }} - \frac{{V_{READ} }}{{40 \times R_{LRS} }} < I < \frac{{V_{READ} }}{{0.926 \times R_{LRS} }}} \\ \end{array}$$

From Eq. (5), the range of I can be roughly calculated as 0.906 I_IDEAL_–1.080 I_IDEAL_. Since I is proportional to the result of multiplying the input by the weight, we can deduce the relative error of the weights approximately in the range of 0.906–1.080. Considering the resistance variation of the memristors, we added ± 10% random noise to the weights during training to improve the generalization ability and robustness of the algorithm.

**Figure 8:**
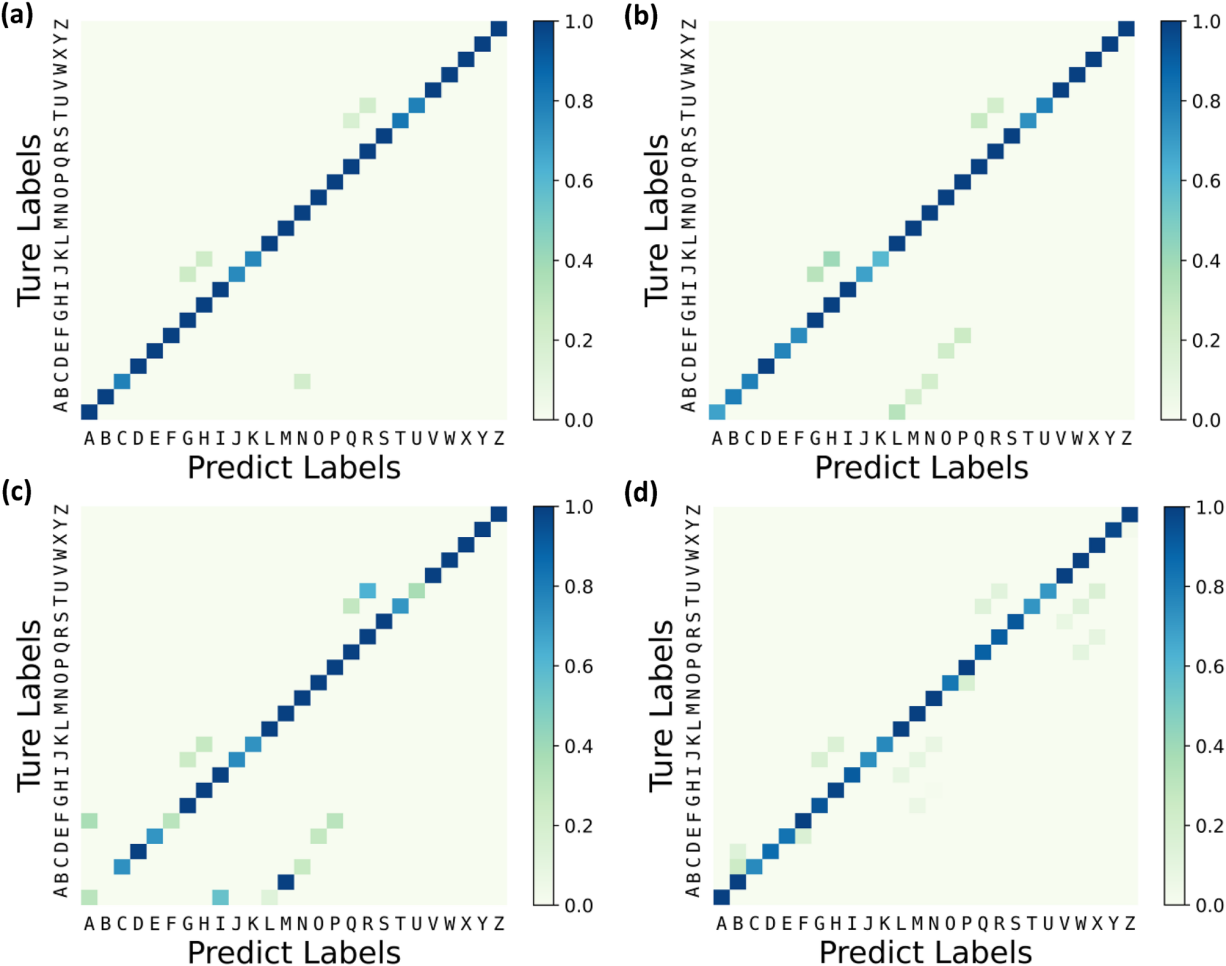
Confusion Matrix of the 26-alphabets recognition by the E-skin system for various scenarios: (**a**) without weight noise, (**b**) with ± 10% weight noise, (**c**) with ± 20% weight noise, and (**d**) measurement result.

After the completion of network training, BANN Braille recognition algorithm with ideal weight achieved a recognition accuracy of 96.53%, the heatmap of recognition result is shown in Fig. 8a. Considering the weight error caused by the variation of the memristor resistance, we added ± 10 and ± 20% random noise to the weights in the test, respectively. The heatmap of the recognition result are shown in Fig. 8b and c. Finally, 1000 samples in the test set were examined, and the result is shown in Fig. 8d. An average recognition accuracy of 91.25% was achieved. Table 1 presents a comparison between a previous work reported in the literature ^11^ and this work in terms of network structure, hardware platform, number of parameters and accuracy for Braille recognition. It is worth mentioning that the recognition accuracy of Ref.^11^ was 92.5%, but the algorithm mapping needed to be conducted on a GPU platform with a very large amount of parameters.

**Table 1:** Comparison between a previous work reported in the literature and this work.

	Network structure	Hardware platform	Number of parameters	Braille recognition accuracy (%)
Ref.^11^	Multilayer Perceptron	GPU	50,432@floating32	92.5
This work	B-FC	Memristor-based hardware	988@binary and 32@10-bit integer	91.25

This work implements the PDMS-based binary braille recognition system on the board-level circuit, providing support for the fabrication of an application-specific integrated circuit for the braille recognition system. If we use 988 memristors to map all the weights of the binary braille recognition algorithm, the scale is very large and difficult to do for a board-level circuit. For the miniaturization of the system, we use the method of time-division multiplexing to map the weights; therefore, the endurance of the memristor will inevitably limit the service life of the system. In this work, we address this issue by replacing the memristor device in the system before the memristor reaches its durability limit. In addition, the system is still relatively bulky. Therefore, in future research, we will focus on integrated circuit-level braille recognition systems, using larger memristor arrays to map network weights and solving the problem of system durability caused by the limited number of erasing and writing times of memristors. Furthermore, we will investigate flexible sensors with higher sensitivity and better stability, and implement the system on a flexible PCB to achieve a more miniaturized, more portable, and more durable wearable braille recognition system.

## Conclusion

In conclusion, an intelligent Braille recognition system based on flexible E-skin and memristive neural network was demonstrated with a good Braille recognition accuracy of 91.25%. The E-skin was constructed with PDMS-based flexible pressure sensors. Braille recognition was realized with a binary neural network based on memristors. The binary neural network algorithm had only two bias layers and three fully connected layers. Such neural network design remarkably reduced the calculation burden and thus the system cost. This work demonstrates the possibility of realizing a wearable and low-cost intelligent Braille recognition system.

## Data Availability

The data that support the findings of this study are available from the corresponding author upon reasonable request.
